# OCCUPATIONAL PERFORMANCE PRIORITIES AND OUTCOMES IN LONG-TERM TRANSITIONAL REHABILITATION FOR YOUNG ADULTS WITH ACQUIRED BRAIN INJURY

**DOI:** 10.2340/jrm.v58.45144

**Published:** 2026-06-04

**Authors:** Charlotte HEURLIN, Marie-Louise SCHULT, Aniko BARTFAI, Annelie ROSÉN, Jan EKHOLM, Monika LÖFGREN

**Affiliations:** 1 Department of Rehabilitation Medicine, Danderyd University Hospital, Stockholm; 2 Department of Clinical Sciences, Karolinska Institutet, Danderyd University Hospital, Stockholm, Sweden

**Keywords:** rehabilitation, acquired brain injury, comprehensive rehabilitation, young adults, Canadian Occupation Performance Measure (COPM), Glasgow Outcome Scale-Extended interview (GOSE)

## Abstract

**Background:**

In a new long-term transitional rehabilitation service following acquired brain injury, occupational performance problems are analysed.

**Aim:**

The objective was to describe these problems and the participants’ prioritization, and also to describe the changes between admission and discharge. Another objective was to describe changes in assessed independence-related disability.

**Design:**

Observational clinical study.

**Patients:**

Young adults (18–25 years), (*n* = 46), who had earlier undergone traditional medical rehabilitation following acquired brain injury.

**Methods:**

The Canadian Occupational Performance Measure (COPM) was used on both admission and discharge. Independence-related general disability was assessed using the Glasgow Outcome Scale–Extended (GOSE).

**Results:**

Patients identified 122 occupational performance problems as important. Most problems were in socialization (3/7), community activities (1/3), and finding paid or unpaid work (1/4).

Self-rated levels of performance and satisfaction in COPM were significantly higher on discharge compared with admission. The range of GOSE scores increased from 4–6 to 4–8 between admission and discharge.

**Conclusion:**

Young adults with acquired brain injury tend to prioritize rehabilitation goals to reduce difficulties in establishing relationships and/or in professional life. On discharge, ratings of implementation and satisfaction of performance were distinctly elevated, while assessed global disability was decreased.

**Clinical rehabilitation impact:**

The results highlight the need for transitional rehabilitation for patient-centred remediating measures.

## LAY ABSTRACT

During the period between 18 and 25 years of age, independent living, working life, and relationships are established. The period is demanding, even without cognitive impairments, and young adults with cognitive impairments due to a brain injury have problems, e.g., engaging in meaningful daily activities and professional development. A new type of long-term (7 years) transitional rehabilitation service has been established to meet the needs of young adults with brain injury. On admission, the young adults participating in the rehabilitation service identified 122 important activity problems, among which the most important were related to work, school, and leisure. The performance of the activities was rated as impaired, and satisfaction with performance was rated as low. On discharge, the ratings had improved. The young adults had developed in a positive manner during these years, but there is no definite conclusion as to what extent this improvement is due to normal development or the rehabilitation interventions.

Both childhood and adolescence involve elements of biological growth and major social role transitions. The transitional period of adolescence to adulthood requires the acquisition of a variety of new, more advanced, and complex skills. This is a period to establish independent living, working life, and relationships. The period of being a young adult – between 18 and 25 years – is demanding, even without cognitive impairments (1). The consequences of an acquired brain injury (ABI) in young adulthood can significantly disrupt key developmental milestones, such as attending college and launching a career (2–4), or establishing independent living and relationships.

Although several studies identified the special needs of adolescents with ABI (5, 6), the focus of rehabilitation efforts is on improving body function and environmental factors. The only aspect of participation discussed in these studies is related to school. Minimal attention and effort are directed towards community participation (5).

Another issue in the transition phase at age 18 is to adjust to the diversity in organization of adult health, community, and educational/work services. Patients and their next of kin often experience considerable frustration while trying to identify the relevant services and find service providers well informed in ABI (7). Young adults with so-called “non-visible disabilities”, i.e., those with disturbances in executive function, attention, memory, behavioural adjustment, and emotional communication, are particularly prone to experience serious obstacles (8).

To address these obstacles, young adults with ABI need the support of professional competence specialized in brain injury rehabilitation. Case management is evolving rapidly; however, according to a recent review (9) the method has some drawbacks. The International Paediatric Brain Injury Society advocates for long-term holistic family-centred support, education for all involved in their care, and improved collaboration across the care pathway to ensure coordinated and effective provision of services (10).

The transitional rehabilitation service for young adults in the present study was created to fill the gap between acute rehabilitation and ordinary health and community services providing patient-centred rehabilitation/support during 7 years, whenever needed. The present type of patient-centred rehabilitation service is very rare. A recent study (11) found less than a handful of services worldwide providing this kind of support.

Active participation by patients is an important aspect of rehabilitation quality as conceptualized in patient-centred practice. A recommended outcome measure for enhancing patient-centred practice is the Canadian Occupational Performance Measure (COPM) (12–15), and studies show that the identification of self-perceived occupational performance problems seems to enhance client motivation and the relevance of individualized goals in rehabilitation.

A new type of long-term transitional rehabilitation service has been established, and one of the aims of the present study was to describe the occupational performance problem choices young adults with acquired brain injury (ABI) made on admission within the fields of “Self-care”, “Productivity”, and “Leisure” using the Canadian Occupational Performance Measure. The second aim was to describe admission–discharge differences in self-selected performance and satisfaction, while the third was to describe assessed independence-related disability.

## MATERIALS AND METHODS

This is a descriptive cohort study.

### Study setting

*Description of the transitional rehabilitation programme.* Needs and preconditions differ for children and adult rehabilitation after ABI. In Sweden, rehabilitation after ABI is organized in several ways. Up to 18 years, the affected children are connected to multidisciplinary teams focusing on development, school, and family relations. Upon turning 18, they are discharged from child rehabilitation services.

For adults, health and community services are more diversified. Patients with ABI requiring substantial assistance and support are granted help separately (https://www.habilitering.se/language/engelska/), while young adults with moderate or mild impairment after ABI are referred to different health and community services, who provide assistance for independent housing (with or without support), higher level studies and career choices, and healthcare for chronic residual symptoms.

To bridge the gap between needs and services for young adults (18–25 years) with moderate or mild residual symptoms after ABI, a specialized team was established, focusing on transitional issues. The interdisciplinary team started in 2012 at the Department of Rehabilitation Medicine, Danderyd University Hospital, Stockholm, Sweden. The team consists of a physician, occupational therapists, neuropsychologists, social workers, speech and language therapists, and physiotherapists. Intensive periods of rehabilitation (about 2–3 appointments/week for 5–6 weeks) are interspersed with periods of sparse contact. When needed, patients can contact the team until they are 25 years old.

The team provides team-based interdisciplinary rehabilitation grounded in evidence-based methods (16) based on the American Congress of Rehabilitation Medicine (ACRM) manual (17), comprising a period of frequent sessions and assistance and support when required, in external contacts with other agencies concerning living conditions, educational, and work choices. Furthermore, the team cooperates with other organizations, such as educational services, housing and community services, and workplaces during work training.

For patients with mild or moderate impairments, interventions in occupational therapy were based on goals identified using the Canadian Occupational Performance Measure (COPM) (15). The treatment plan was uniquely focused on those occupational issues that the client identified as high in importance and low in performance and satisfaction, often using occupation-based strategy training influenced by the method of Cognitive Orientation to Daily Occupational Performance (CO-OP) (18).

For patients with more severe disability, the interventions focus on adjusting the environment and enabling engagement in preferred activities according to selected goals in COPM. There is also an emphasis on informing and educating the clients’ network and supply of official documentation based on detailed team-based assessments, which require access to other societal support services.

On discharge, at the age of 25, the patients are offered closing contact with the team, including a detailed status assessment, a concluding conference to discuss future possibilities, and written and verbal information on the different types of societal services.

### Participants

We are presenting data from a large collection based on clinical data from 9 January 2012 to 8 January 2021, including patients (*n* = 46) with data in COPM from both admission and discharge (up to 7 years later). Thus, many of the patients are still in the programme and due to the lack of discharge data they are not included in the study.

Inclusion criteria were those living in the Stockholm Region and affected by ABI after 2 years of age, with symptoms leading predominantly to cognitive and/or behavioural dysfunction, resulting in significant difficulties in establishing independent living and professional life. The patients, here termed young adults, were enrolled in the long-term rehabilitation service between 18 and 25 years of age.

There were two major referral sources: (a) young adults who as children had been in paediatric rehabilitation due to a CNS tumour, stroke, or brain injury and were currently discharged due to their reaching the age of 18 years and (b) young adults from the age of 17–18 years who had undergone acute inpatient rehabilitation in an adult setting, due to stroke and brain injury. Demographic data are presented in Table I.

**Table I:** Demographic data of the patients (*n* = 46) on admission

Demographics	
Gender, *n* (%)	
Male	22 (48)
Female	24 (52)
Age, years*, mean (SD), min–max	21 (2), 18–24
Vocational status, *n* (%)	
Employed	8 (17)
Student	15 (33)
Receiving economic compensation**	23 (50)
Years in education, *n* (%)	
< 9 years	3 (7)
9 years	22 (48)
12 years	14 (30)
> 12 years***	7 (15)
Living conditions, *n* (%)	
With parents	36 (78)
Not with parents	10 (22)
Civil status, *n* (%)	
Single	35 (76)
Having a partner	5 (11)
Living with a partner	6 (13)

* Age at time of enrolment in rehabilitation,

** patients may receive different types of compensation for lack or loss of working capacity,

*** for some ongoing.

### Procedure

Admission to the long-term rehabilitation service was decided by a senior rehabilitation medicine specialist and the rehabilitation team. On admission, the physician collected the clinical and demographic data, including the Glasgow Outcome Scale Extended Interview (GOSE), while a specialist occupational therapist collected data of the COPM. At discharge when the patient reached 25 years of age, data on GOSE and COPM were collected again.

### Assessment instruments

The *Canadian Occupational Performance Measure (COPM)* is a generic instrument (19) used by occupational therapists to measure occupational performance and satisfaction with performance in the areas of self-care, productivity, and leisure. It comprises a semi-structured interview (20) designed to identify problems in occupational performance. It also assists in goal setting and measures change in self-ratings of performance and satisfaction related to self-identified rehabilitation goals over time. During the interview, the patients indicate some activities they experience problems with and would like to improve performance-wise. Treatment planning and the individual rehabilitation goals are based on the indicated problems with the goal to improve performance and satisfaction in selected occupational problem areas and activities. COPM is conducted in 3 stages where the patient (*i*) identifies occupational problems in self-care, productivity, and leisure; (*ii*) prioritises 3–5 of those as the most important rehabilitation goals; and (*iii*) rates the level of current performance (COPM-P) and satisfaction (COPM-S) with the performance for each prioritized problem on a 10-point scale (1 = not being able to do it/not satisfied at all to 10 = being able to do it extremely well/extremely satisfied).

The *Glasgow Outcome Scale – Extended Interview (GOSE)* is a widely used outcome instrument used to assess global disability and recovery in clinical care after traumatic brain injury (TBI) (21). The original Glasgow outcome 5-level scale (GOS) (22) has been further developed by splitting levels 3–5 into 2 levels, each mainly based on dependence/independence, resulting in an 8-point scale (from dead to fully returned to normal life). The criteria defining points 4–8 are described in connection with the improvements (see Results, page 5 and Table IV).

### Descriptive analysis of problems, leading to rehabilitation goals, that were indicated and prioritized by the patients using COPM

The descriptive analysis included a retrospective compilation of the answers in the COPM (performed by 2 of the authors, CH + AR (CH is a specialist OT, MSc, with many years of clinical experience of brain injury rehabilitation; AR is an RTP, PhD experienced in quantitative and qualitative analysis). The number of occupational problems reported were categorized and summed into respective COPM areas (and activities): (a) self-care (personal care, functional mobility, and community management); (b) productivity (paid or unpaid work, household management, play, or school); and (c) leisure (quiet recreation, active recreation, and socialization). To ensure reliability of categorization into respective areas, all authors rechecked the sorting.

### Power analysis

Power analysis was based on the primary outcome variable, defined as the change in the COPM score from admission to discharge. A one‑sample t‑test for within‑subject change was performed to detect a change of 2.2 points (23), assuming a standard deviation of 2.1 points. With a power of 90% and a two‑sided significance level of 0.05, 10 participants were required. Allowing for a potential dropout rate of 20%, at least 13 participants were needed.

### Statistical analysis

Descriptive statistics: the number (*n*), proportion (%), mean (m), standard deviations (SD), and median (md) 25th and 75th percentiles were calculated for demographic data and clinical characteristics. Furthermore, medians and percentiles were calculated on admission and discharge for the COPM occupational performance areas (self-care, productivity, and leisure), including the specific activities connected to each area. To analyse the change between admission and discharge for COPM performance and satisfaction and GOSE, the Wilcoxon signed rank test (within group) was performed. To assess the effect sizes between admission to discharge in COPM and GOSE, the Related-Samples Kendall’s W test was performed. To interpret the magnitude of effect sizes, Cohen’s *d* was used (24), classified as 0.2 (small effect), 0.5 (moderate effect), and 0.8 (large effect). All statistical analyses were performed in SPSS 29 (IBM Corp, Armonk, NY, USA). The significance level was set as *p* < 0.05, two-tailed.

## RESULTS

### Clinical characteristics

Almost half of the young adults had TBI (Table II). Almost half had their brain injury after the age of 18. The median score of general independencies was GOSE level 5 (moderate disability, lower level, which means “unable to work or study, or unable to participate in social and leisure activities” or “having constant problems with family and friendship”). For a definition of GOSE levels, see Table IV.

**Table II:** Clinical characteristics of the patients on admission (*n* = 46)

Characteristics	Subject
Age at injury, mean (SD), min–max OR	16.6 (5.3), (2–23)
Median (25th–75th)	18 (14.8–20)
Age at injury, *n* (%)	
2–5	4 (9)
6–12	3 (7)
13–18	20 (43)
> 18	19 (41)
Diagnosis, *n* (%)	
Traumatic brain injury	21 (46)
Cerebral vascular lesion	8 (17)
Brain tumour	10 (22)
Encephalitis	3 (7)
Anoxia	1 (2)
Concussion	1 (2)
Other	2 (4)
GOSE score, *n* = 44, *n* (%)	
GOSE level 4	4 (10)
GOSE level 5	20 (45)
GOSE level 6	20 (45)
GOSE missing	2 (4)

### Problems selected and prioritized by the patients

On admission, the 46 young adults identified and prioritized 122 important occupational performance problems in the COPM areas of Self-care, Productivity, and Leisure described below and summarized in Table III (see the 2 left columns). The number of problems varied from 1 to 6 per patient.

**Table III:** Number of occupational performance problems (*n* = 122) (left column) in the 3 areas of self-care, productivity, and leisure with their respective activities and number of patients having indicated each problem (and in % of the 46 patients) (second left column). Middle 2 and right 2 columns show self-rating of performance and satisfaction on admission and discharge. A higher score indicates higher performance and satisfaction

COPM areas and their activities	Problems, *n*	Patients *n* (%)	Rating of performance Median (25th–75th)		Ratings of satisfaction Median (25th–75th)	
			Admission	Discharge	Admission	Discharge
Self-care						
Personal care	4	4 (9%)	4 (2–5)	6 (4–8)	1 (1–6)	5 (3–6)
Mobility	7	7 (15%)	4 (3–5)	8 (4–9)	2 (1–4)	8 (6–9)
Community management	19	16 (35%)	5 (4–6)	8 (6–10)	2 (1–6)	8 (5–10)
Productivity						
Paid/unpaid work	14	12 (26%)	5 (4–6)	9 (6–10)	4 (1–5)	10 (7–10)
Household management	10	10 (22%)	4 (4–5)	9 (7–10)	4 (1–6)	8 (8–10)
Play/school	16	11 (24%)	5 (4–6)	7 (6–8)	2 (2–6)	8 (6–8)
Leisure						
Quiet recreation	16	13 (29%)	6 (4–7)	8 (6–9)	4 (2–7)	8 (7–9)
Active recreation	14	14 (30%)	4 (3–7)	7 (5–9)	2 (1–6)	6 (4–8)
Socialization	22	20 (44%)	5 (3–6)	8 (8–9)	2 (1–4)	9 (7–10)

*Self-care.* The patients selected 30 problems in the area of Self-care including the activities of Personal care (*n* = 4), Mobility (*n* = 7), and Community management (i.e., manage activities in society) (*n* = 19). *Personal care* (the problem “manage the morning routine”) was indicated by 4 patients (9%). Problems in *Mobility* (chosen by 15%) were such as “keeping balance during walking on varied terrain”, “avoiding bumping into things”, and “not being able to run”. The problems in the field of *Community Management* were chosen by 16 patients (35%) who, e.g., reported problems regarding control over their own economy, and using and coping with public transportation and with strong, intrusive stimuli, such as loud noises or overwhelming visual environment.

*Participation and activities in society and at home – “Productivity”.* The patients indicated 40 problems within the area of Productivity. Fourteen problems were selected in *Paid/unpaid Work*, where problems related to work dealt with “finding and keeping a job” (chosen by 26% of the patients). Ten problems were selected in *Household Management*, such as “planning, organising, and initiating household activities”, chosen by 10 patients (22%). Finally, in the field of *Play/School*, 16 problems were selected; those related to school were the “need for more time when performing tasks”, “reading impediments”, “remembering information”, and “need for study support”.

*Leisure.* The patients indicated 52 occupational performance problems in the area *Leisure*, including the activities in *Quiet Recreation* (*n* = 16), *Active recreation* (*n* = 14), and *Socialization* (*n* = 22). Sixteen patients (28%) chose the problems in *Quiet Recreation* and reported problems with the “energy to read a book with concentration on following the lines”, “watching a film with satisfaction”, and “computer gaming”. In *Active Recreation* (chosen by 30%), the patients had problems with “initiating various physical activities and hobbies”. In the field *Socialization* (chosen by 44%), the patients had problems with “being spontaneous”, “socializing with friends and relatives”, “maintaining focus in a conversation with several people”, or “talking on the phone and e-mailing”.

### Ratings on admission and discharge regarding performance and satisfaction

**Fig. 1:**
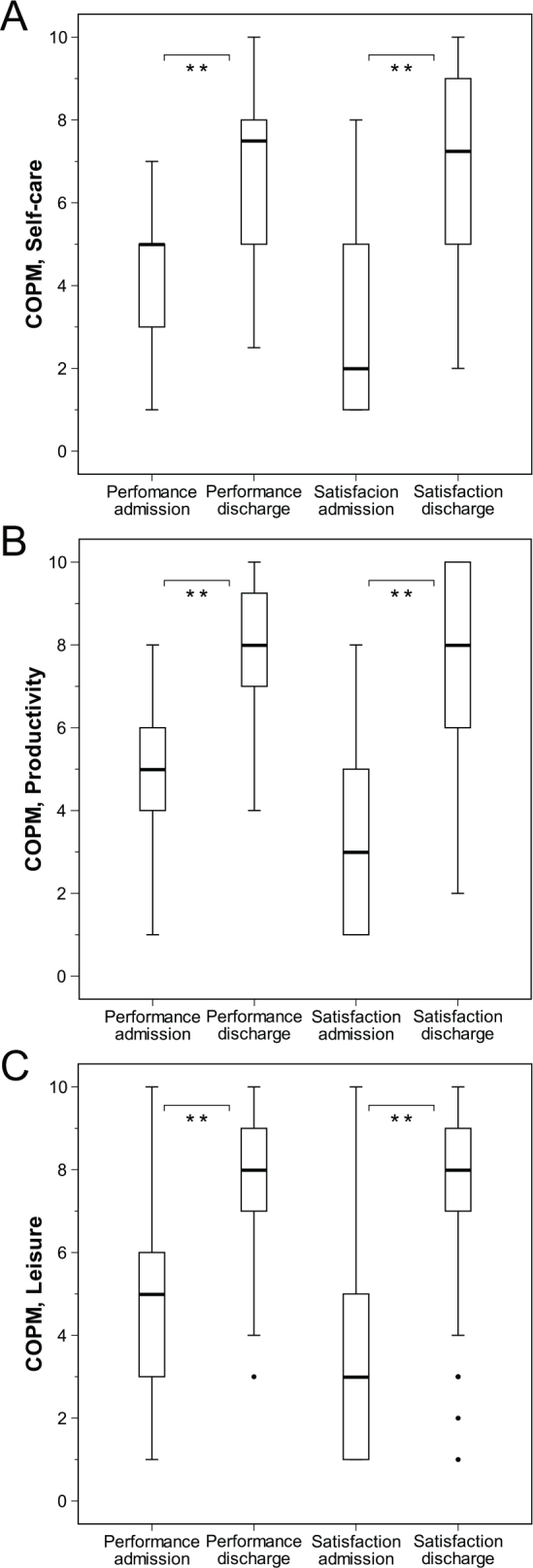
**Improved performance and satisfaction in the areas of self-care, productivity and leisure.** Boxplots showing improvements of medians and percentiles between admission and discharge in patients’ rating of COPM performance and satisfaction in the areas of (A) self-care, (B) productivity, and (C) leisure. ** = significant difference *p* < 0.001, in Wilcoxon signed ranks test and Related-Samples Kendall’s Coefficient of Concordance. A higher score indicates higher performance and satisfaction.

The trend regarding the patients’ ability to conduct the selected activities on admission and discharge is shown in a boxplot (Fig. 1: Improved performance and satisfaction in the areas of self-care, productivity, and leisure). There were significant improvements (*p* < 0.001) in each COPM area (self-care, productivity, and leisure) with large effect sizes varying from 0.707 to 0.903 within the different areas for both performance and satisfaction (Fig. 1).

Part of the data behind Fig. 1 can be seen in Table III (middle 2 and 2 right columns), which provides data on the young adults’ ratings on admission and discharge regarding performance and satisfaction with their ability to conduct the selected activities. As an example of significant improvement, the activity “Paid and unpaid work” increased from 5 to 9 in performance from admission to discharge and from 3.5 to 9.5 in satisfaction (where 10 is the highest possible). Another example involves the ratings of “socializing”, including among other things “socializing with friends and relatives”, which showed an increase from 3.8 to 7 in performance and from 2 to 9 in satisfaction.

The overall results for COPM show that the patients improved in COPM-P and COPM-S with large effect sizes, namely > 0.8 (*p*-value < 0.001) from admission to discharge.

### Assessment according to the GOSE 8-scale

Overall, the patients increased statistically significantly in GOSE from admission to discharge, although the effect size was considered as small, *d* = 0.336 (Table IV).

**Table IV:** Distribution of patients in GOSE scale steps assessed by a physician on admission and on discharge and results of statistics tests of admission vs discharge (*n* = 41*)

GOSE Scale step	Admission Frequency (%)	Discharge Frequency (%)	Test statistics
4: Severe disability, upper level**	3 (8)	2 (5)	
5: Moderate disability, lower level	19 (46)	10 (24)	
6: Moderate disability, upper level	19 (46)	18 (44)	
7: Good recovery, lower level		9 (22)	
8: Good recovery, upper level		2 (5)	
Total	41 (100)	41 (100)	
Min–max	4–6	4–8	
Median (25th–75th)	5 (5–6)	6 (5–7)	
Z (*p*-value) **			–3.3 (< 0.001)
Kendall’s W (*p*-value) ***			0.336 (< 0.001)

Improvement in assessed disability between admission and discharge.

Higher scores indicate a more favourable disability level.

* Patients with data on admission and discharge.

** The original scale steps are split into 2 sub-levels: lower and upper.

GOSE 8-scale point 4 is defined as “function in home: unable to look after themselves for 8 hours; function outside home: unable to shop or unable to travel” (21). Three young adults were assessed to be at that level of dependence/independence (see Table IV). On admission, 19 patients were assessed to scale point 5, which means “unable to work or study, or unable to participate in social and leisure activities” or “having constant problems with family and friendship”. Moreover, almost half of the patients (46%) were assessed on admission to be at scale point 6, defined as “reduced work capacity or much reduced participation in social and leisure activities or frequent problems with family and friendships”. On discharge, 9 young adults were assessed to scale point 7, which is defined as “participating a bit less in social and leisure activities or occasional problems with family and friendships”, while 2 patients reached scale point 8, i.e., “fully returned to normal life” (21).

## DISCUSSION

This is the first study describing a unique rehabilitation approach, transitional rehabilitation addressing the specific needs of young adults with ABI in supporting them to develop life skills to achieve their goals. These actions are grounded in the issues identified jointly by the patient and the OT during the initial COPM interview. The main result of the present study is the detailed description of occupational performance problems prioritized by importance by the young adults. Presently, there is a lack of such descriptions and therefore it is a specific contribution of this study.

### Occupational performance problems in comparison with other groups of young adults

The most frequently indicated important occupational performance problems were within “Socializing” (within the Leisure domain), entailing occupations such as “socializing with peers” and “socializing with friends and family”. For some young patients, these fields included initiating relationships. “Socializing” is also a typical age-related problem in healthy undergraduates (25) and is part of normal development. However, ABI leading to executive and communicative impairments (26) counteracts the developing skills and underlines the importance of the transitional team’s professional influence during this period.

Within the domain of Productivity, “paid or unpaid work, or seeking employment” was also frequently indicated as an important occupational problem. Likewise, the occupational problem “studying” was indicated by several. Similar results are presented in a study of young war veterans (27), aged 22–29 years, where the most important occupational performance problems reported were productivity and school challenges (transition into student role), leisure (occupations such as socializing and participation in relationships), and self-care (eating and weight gain, managing sleep disruption).

Some of our patients have indicated a severe problem in “keeping control over own economy” in the domain of Self-care. The reason why “taking care of household” was not more frequently noted in the present study may be that 78% of the patients were living with their parents and thus did not need to assume the complete responsibility for household management and could focus on other activities. These results are comparable to a study of young adults suffering from physical disabilities where the majority were living with their parents (28). Several of our patients identified performance problems in “using public transportation”, and this was also identified in a systematic review (29) of factors affecting driving and public transportation among youth and young adults with acquired brain injury.

Thus, the occupational performance issues found in the young adults are in general related to productivity and leisure as they are within a working or studying age group. The results may be seen as a sign of orientation towards highly relevant problems, preparing for adult working life and socializing with other people.

### Occupational performance problems in comparison with other age and diagnostic groups

However, when comparing the results of young adults suffering from ABI in the present study with other age groups and diagnostic groups, the patterns of important occupational issues differ. Enemark Larsen et al. (30) compared 4 different cohorts, and found differences in the distribution of occupational performance issues. The older the patient, the more prominent the performance issues in Self-care, especially regarding inpatients. For patients of working age, Productivity had most performance issues. The same was true for middle-aged persons with persisting symptoms following a mild TBI (31). Leisure was an important occupational issue within the knee-replacement and hand-surgery cohorts (30) and after mild TBI (31). In these cohorts it seems that both age and diagnoses can contribute to different performance issues.

### Before-and-after comparisons

The present results showed statistically significant improvements from admission to discharge in all 3 problem areas, i.e., self-care, productivity, and leisure, both in performance and satisfaction with large effect sizes. We found no studies with similar cohort for comparisons with our COPM results. There are post-intervention results from a small study of young adults with other neurological disabilities after participation in vocational rehabilitation (28). These patients experienced fewer problems and showed improved occupational performance in work, as well as self-care and leisure and improved satisfaction with performance. Another study of occupational performance goals regarding children with executive function deficits after acquired brain injury (ABI) showed that perceived occupational performance and satisfaction improved significantly for the trained goals (32).

In the present study, the original version of the COPM was used; however, recent work has improved and deepened the analysis of the instrument (12). The COPM is only one way of describing the patient’s occupational performance, how they prioritize goals for improvement, and how performance and satisfaction may improve overtime. Other measures investigate occupational performance, patterns of daily occupations, and occupational balance. These aspects have been found to be related to health and well-being (33–36).

When using COPM for intervention evaluation, a relevant change score must be defined for the present group of patients. A 2-point threshold has occasionally been cited as representing a clinically important change (15). However, the literature demonstrates considerable variability in estimates of clinically important change, ranging from 0.9 to 3.5 or more (37). We selected a difference of 2.2 points as the expected change score (23), since this change score is in the middle area of the values presented in the literature.

The median level of GOSE ratings was 5 on admission, meaning “unable to work or study, or unable to participate in social or leisure activities” or “having constant problems with family and friendships” (21). No study was found with the same type of adolescents and young adult patients, e.g., none in the acute or subacute situation but in a later phase of rehabilitation need. Nevertheless, for comparison, we noted that in a study of adults with severe traumatic brain injury, the mean GOSE score at the 3-month follow-up was 5 (38).

The GOSE is a measure of global disability and recovery after traumatic brain injury and by extension an indicator of dependence or independence in daily living. In a recent study (39), it was found to be 1 of the 3 reliable predictors of outcome after ABI. The other 2 predictors were functional outcome measured by the Functional Independence Measure (FIM) and the presence of chronic fatigue. The GOSE is the most frequently used simple outcome measure (21) despite the criticism, as it provides a relatively stable course outcome of recovery. A major advantage is that, based on the formalized, comprehensive interview, it covers a wide scope of aspects in daily living and thus represents a reliable estimate. A frequent disadvantage in application is, however, differences between raters (21). In the present study, the ratings were administered mostly by 1 rater, a physician specialist in PRM, increasing the reliability of the data.

The comparison between admission and discharge showed significant improvements on discharge. The median score of all on discharge was 6; 9 patients had reached a score of 7, and 2 patients achieved a score of 8 (“fully returned to normal life”). Although the changes in scores are modest, these changes probably meant for the participants considerable functional improvements towards living an independent life. As mentioned earlier, studies with comparable data are scarce. A recent study (40) covering the Danish register for young individuals with acquired brain injury indicated a comparable distribution of GOSE scores, and a study of adults with moderate traumatic brain injury (41) also showed ratings of a comparable order of magnitude at a 12-month follow-up. The latter patient group is very different in many aspects, but a tentative hypothesis might be that the similarities in the level of dependency/independency reflect the distribution of residual effects of ABI in general.

### Methodological issues

The study found considerable differences in effect size between COPM and GOSE. The common aim of both GOSE and COPM is to describe the functional residual effects of a brain injury. However, these 2 measures differ in which professional context they are used. GOSE is the preferred measure in the medical context, such as within neurotrauma or by specialists in rehabilitation medicine, “characterizing TBI outcomes solely from the perspective of investigators and clinicians” (42). COPM, on the other hand, is an instrument for occupational therapists including both professional and patient perspectives on performance and satisfaction. Another measure of functional status, the Functional Status Examination (FSE) (43), also carried out by occupational therapists, was compared with GOSE (44) and the authors found that “the GOSE is a coarser representation of function than the FSE”. GOSE and COPM have not been compared yet, but we suggest that COPM also captures a more nuanced characterization of outcome.

This clinical study represents a subgroup of patients from a large ongoing data collection, those with data from both inclusion and discharge. The major limitation of this clinical study is the lack of a comparison group. Also, the cohort consists of 2 subgroups: those injured at a younger age and those who were injured in their upper teens. Thus, no conclusion can be drawn regarding age effects.

Another limitation is that data were collected during the first 9 years of the team’s operation. Thus, the clinical experience has grown, and interventions have become more sophisticated during these years. Among others, evidence-based processes based on the ACRM manual (17) were introduced generally in the department of rehabilitation, which most likely also influenced the methods and procedures used by the team.

Among the strengths of the study is the representativeness of the sample. All eligible young individuals following ABI in the greater Stockholm area were referred to the team. Also, the staffing was relatively unaltered during these years, increasing the reliability of the ratings.

Thus, the developmental period between 18 and 25 years constitutes a major milestone in personal growth for everyone. Follow-up studies of groups after childhood ABI found somewhat conflicting data. Some describe a rather satisfactory level of participation in adulthood but with some negative influences on quality of life, while other studies described limitations in participation (45). Residual symptoms, such as fatigue, may also influence the level of participation (8). COPM and GOSE data in our study showed a positive development, but our study lacks a comparison group; thus, we cannot draw a direct conclusion regarding treatment effects for this group of patients. Controversies in earlier results might also be due to differences in the type and extent of ABI in the investigated groups. The participants in our study had limited motor and varying degree of cognitive impairments, allowing more leeway for psycho-educative and psychosocial interventions. Hence, we venture to conclude that our transitional rehabilitation team has contributed to this positive development and prevented stagnation for our participants.

### Conclusion

Young adults with acquired brain injury in the present cohort tend to prioritize rehabilitation goals to reduce difficulties in establishing relationships and/or in professional life. Self-selected performance and satisfaction of performance were distinctly elevated on discharge. Assessed global disability was decreased on discharge.

## Data Availability

The data associated with the paper are not publicly available but are available from the corresponding author on reasonable request.
